# Calcified sewing needle as a urinary foreign body in a pediatric patient: a case report

**DOI:** 10.1097/RC9.0000000000000249

**Published:** 2026-02-10

**Authors:** Yalda Obaidy, Ajmal Sherzad, Dunya Moghul

**Affiliations:** a Department of Surgery, Ataturk Hospital, Kabul, Afghanistan; b Independent Researcher

**Keywords:** bladder foreign body, bladder stone, case report, cystolithotomy, pediatric urology

## Abstract

**Introduction and Importance:**

Foreign bodies (FBs) in the bladder can result from iatrogenic causes, migration from adjacent organs, penetrating injuries, or self-insertion. They serve as a nidus for calcification and stone formation. While bladder stones related to FBs are occasionally seen in adults, such cases are extremely rare in children.

**Presentation of Case:**

A 2.5-year-old girl (10 kg) presented with chronic irritability, abdominal pain, dysuria, and intermittent hematuria for 1 year, unresponsive to antibiotics and antipyretics. Examination was unremarkable, with a leukocyte count of 9900. Urinalysis showed microscopic hematuria and pyuria. Ultrasound revealed a 33 mm echogenic structure in the bladder, which was confirmed by a kidney, ureter, and bladder (KUB) radiograph as a calcified needle. No history of trauma or abuse was reported, and the child’s development was normal. Open cystolithotomy was performed, successfully extracting the needle. Postoperatively, a urethral catheter was retained for 2 days and antibiotics were administered. The patient was discharged in good condition on day 3.

**Clinical Discussion:**

Bladder FBs in children are rare and present with nonspecific urinary symptoms. Imaging is essential for diagnosis, while management depends on the characteristics of the object and available resources. Endoscopic removal is ideal, but open surgery may be necessary in resource-limited settings or when dealing with rigid, sharp objects.

**Conclusion:**

Prompt recognition and early imaging are essential in pediatric bladder FBs. Timely surgical management prevents avoidable complications that LMIC health systems are less equipped to manage once advanced disease develops.

## Introduction

Foreign bodies (FBs) within the bladder may be introduced iatrogenically, by migration from an adjacent organ, through penetrating injury, or through self-insertion. FBs provide an ideal nidus for calcification and stone formation, and they are responsible for a significant proportion of bladder stones in females. However, such cases are exceedingly rare in childhood^[^1^]^. This case highlights delayed diagnosis and limited pediatric urologic resources in low-resource settings, underscoring the importance of early recognition to prevent complications.

This manuscript has been reported in line with the SCARE criteria^[^2^]^.

## Presentation of case

A 2.5-year-old girl weighing 10 kg presented to the hospital with chronic symptoms persisting for 1 year, including irritability, abdominal pain, dysuria, and intermittent hematuria. These symptoms were refractory to ongoing medication, including antibiotics and antipyretics.

HIGHLIGHTS

- Chronic urinary symptoms in young children should prompt evaluation for rare causes, including intravesical FBs, even when history is unclear.
- FBs may become calcified when retained long-term in the bladder, potentially mimicking stones on imaging.
- Ultrasound and KUB radiography together provide reliable identification and characterization of radiopaque bladder FBs.
- Open cystolithotomy remains an effective and safe approach for removing large or calcified foreign objects in pediatric patients.
- A multidisciplinary assessment, including evaluation of safeguarding concerns, is essential when the mechanism of FB insertion is unexplained.

**Figure 1.**
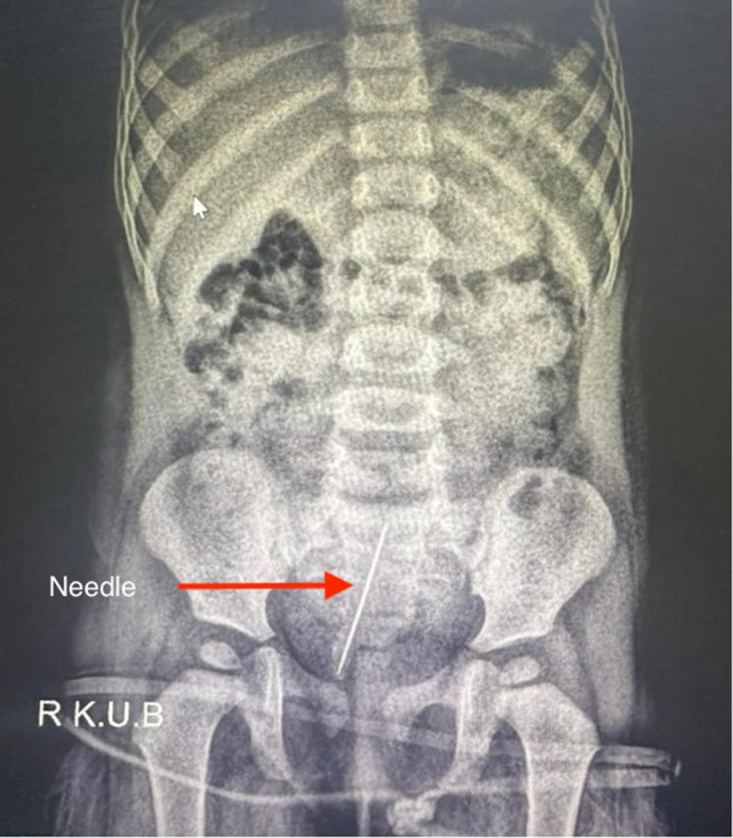
Kidney, ureter, and bladder (KUB) radiograph showing a linear radio-opaque foreign body (arrow) within the urinary bladder, consistent with a calcified sewing needle. The increased density at one end reflects mineral deposition from prolonged urine exposure.

**Figure 2.**
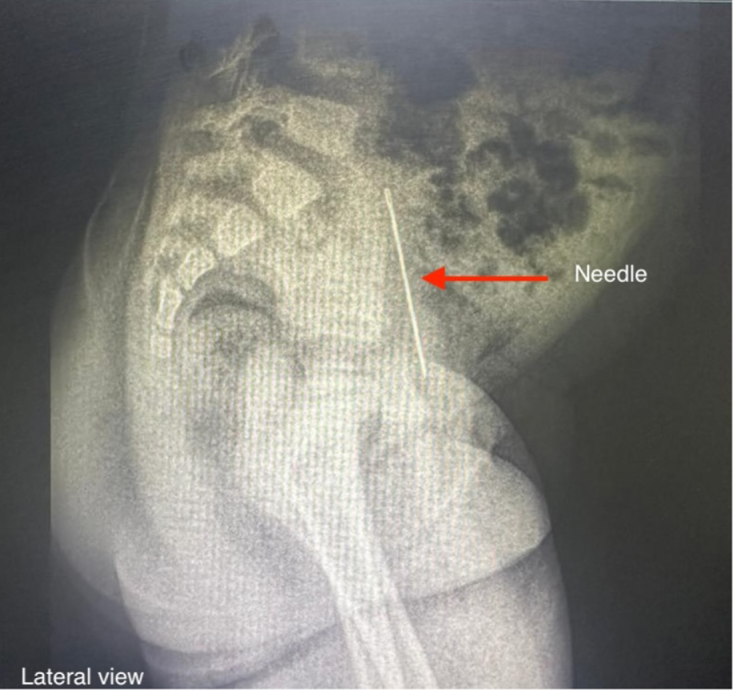
Lateral radiograph demonstrating the intravesical sewing needle (arrow), with clear visualization of its position, orientation, and relationship to adjacent pelvic structures.

Physical examination was unremarkable, and the leukocyte count was 9900. Urinalysis revealed 23–25 erythrocytes and numerous leukocytes per high-power field on microscopic examination. Diagnostic imaging, including ultrasonography, revealed an echogenic linear structure approximately 33 mm along the right lateral wall of the bladder. A kidney, ureter, and bladder (KUB) radiograph confirmed the presence of a linear, radio-opaque FB within the urinary bladder, consistent with a needle. The FB demonstrated increased density at one extremity, indicative of calcification, likely due to mineral deposition from prolonged exposure to urine (Figs 1 and 2).

The family denied any domestic violence and was unaware of how the needle entered the child’s body. The child’s mental status appeared normal, with all growth and developmental milestones appropriate for age.

**Figure 3.**
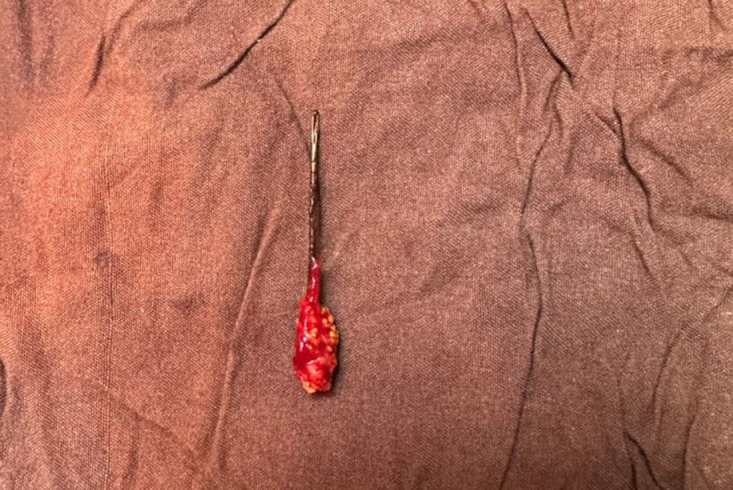
Intraoperative photograph of the extracted sewing needle, showing significant surface calcification and encrustation, confirming long-standing retention in the bladder.

Open cystolithotomy under general anesthesia was performed to extract the calcified needle from the bladder (Fig. 3). An indwelling urethral catheter was left in place for 2 days. Appropriate antibiotic therapy was initiated based on positive urinalysis and culture results.

The patient was discharged in good condition on the third postoperative day. At the 1- and 3-month follow-ups, the patient continued to be free of urinary symptoms, with no reported issues such as dysuria, frequency, or urgency.

## Discussion

FBs in the urinary bladder of children are rare occurrences but pose significant diagnostic and therapeutic challenges. These cases often present with nonspecific lower urinary tract symptoms such as dysuria, hematuria, urinary frequency, pelvic pain, or urinary retention. The nonspecific nature of these symptoms can make diagnosis difficult, particularly in pediatric patients^[^3,4^]^, necessitating a high index of suspicion for accurate diagnosis. Imaging plays a pivotal role in identifying and characterizing the FB. Plain radiographs are effective for detecting radiopaque objects, while ultrasound is valuable for identifying radiolucent materials and assessing associated complications such as bladder wall thickening or stones. In more complex cases, advanced imaging techniques like CT scans may be required to determine the size, shape, location, and potential complications of the FB^[^5^]^.

The etiology of FBs in the bladder varies widely and can include self-insertion, iatrogenic causes, migration from adjacent organs, or trauma. Self-insertion is often driven by curiosity or psychological factors in children, while iatrogenic causes may involve retained surgical instruments or catheter fragments. Migration from adjacent organs can occur due to conditions such as fistulas or perforations, and trauma may result from penetrating injuries^[^6^]^.

Treatment approaches for urinary bladder FBs depend on several factors, including the object’s size, shape, location, mobility, and the resources available at the healthcare facility. Endoscopic removal is the preferred method in most cases as it minimizes trauma and allows for quicker recovery. Techniques such as transurethral cystolitholapaxy or holmium laser lithotripsy are commonly employed when feasible^[^7,8^]^. However, in resource-limited settings where endoscopic equipment may not be available or when the FB is large or rigid with sharp edges that pose a high risk of mucosal injury during extraction, open surgical approaches like cystolithotomy become necessary^[^9^]^. In our case, treated at a governmental hospital in Afghanistan, an open cystolithotomy was performed because resource limitations and the absence of endoscopic equipment made minimally invasive options unavailable^[^10^]^. This approach aligns with recommendations for cases where minimally invasive techniques are not feasible.

Postoperative care is critical to ensure a smooth recovery and to monitor for potential complications such as infections, urethral strictures, or bladder dysfunction. Long-term follow-up is particularly important in pediatric patients to prevent late sequelae and to evaluate for any recurrence of symptoms or the reintroduction of FBs^[^11^]^. Caregiver education and improved household safety measures are essential to prevent accidental insertion or exposure to FBs in young children.

Although considerations were raised regarding possible child abuse, Afghanistan currently lacks formal pediatric social service systems for further assessment. Without established evaluation processes, no additional conclusions can be drawn, and our discussion is limited to acknowledging this context.

In summary, pediatric bladder FBs require a high index of suspicion due to nonspecific urinary symptoms. Basic imaging modalities such as ultrasound and plain radiography are effective first-line tools for diagnosis, particularly in low-resource settings. Surgical management should be individualized based on the FB characteristics and available resources, with open cystolithotomy remaining a safe and effective option when endoscopic facilities are unavailable. Increased caregiver awareness and community education are essential to reduce delayed presentation and prevent similar incidents in children.

## Conclusion

Prompt recognition and early imaging are essential in pediatric bladder FBs. Timely surgical management prevents avoidable complications that LMIC health systems are less equipped to manage once advanced disease develops.
